# Excess Mortality Rate During Adulthood Among Danish Adoptees

**DOI:** 10.1371/journal.pone.0014365

**Published:** 2010-12-16

**Authors:** Liselotte Petersen, Thorkild I. A. Sørensen, Erik Lykke Mortensen, Per Kragh Andersen

**Affiliations:** 1 Institute of Preventive Medicine, Copenhagen University Hospital, Copenhagen, Denmark; 2 National Centre for Register-based Research, University of Aarhus, Aarhus, Denmark; 3 Institute of Public Health and Center for Healthy Aging, University of Copenhagen, Copenhagen, Denmark; 4 Department of Biostatistics, University of Copenhagen, Copenhagen, Denmark; University of Otago, New Zealand

## Abstract

**Background and objective:**

Adoption studies have been used to disentangle the influence of genes from shared familial environment on various traits and disease risks. However, both the factors leading to adoption and living as an adoptee may bias the studies with regard to the relative influence of genes and environment compared to the general population. The aim was to investigate whether the cohort of domestic adoptees used for these studies in Denmark is similar to the general population with respect to all-cause mortality and cause-specific mortality rates.

**Methods:**

13,111 adoptees born in Denmark in 1917, or later, and adopted in 1924 to 1947 were compared to all Danes from the same birth cohorts using standardized mortality ratios (SMR). The 12,729 adoptees alive in 1970 were similarly compared to all Danes using SMR as well as cause-specific SMR.

**Results:**

The excess in all-cause mortality before age 65 years in adoptees was estimated to be 1.30 (95% CI 1.26–1.35). Significant excess mortality before age 65 years was also observed for infections, vascular deaths, cancer, alcohol-related deaths and suicide. Analyses including deaths after age 65 generally showed slightly less excess in mortality, but the excess was significant for all-cause mortality, cancer, alcohol-related deaths and suicides.

**Conclusion:**

Adoptees have an increased all-cause mortality compared to the general population. All major specific causes of death contributed, and the highest excess is seen for alcohol-related deaths.

## Introduction

The genetic and familial environmental influences creating familial correlations in traits and familial aggregation of diseases may be disentangled by adoption studies. However, when generalizing from adoptees to the general population, it is important whether adoptees are similar to the general population regarding the trait in question. In Danish adoption studies of mortality rate, a moderate genetic influence has been found, in particular indicating genetic effects on cause-specific mortality from infectious and vascular disease [1]–[3]. Hence possible discrepancies of adoptees and the general population regarding mortality and cause-specific mortality rates should be considered. If adoptees differ from the general population, this could be caused by the same factors, including genetic load, leading to pregnancy and adoption, and/or the difference may be an effect of living as an adoptee.

Adoption as a risk factor for mental and behavioral problems has been investigated, but few and contradicting results have been reported on domestic non-familial adoptions [4]. In a longitudinal study comparing domestic adoptees with same sex classmates, more symptoms of maladjustment at age 11 years were found in adoptees, whereas the differences compared to non-adoptees were very small at age 15 years [5]. A similar conclusion was reached in a review; “The increased vulnerability of adopted children is restricted primarily to individuals in the middle childhood and adolescent years” [6]. However, in a Swedish cohort of 7340 adoptees, the rate of suicide before the age 40 years was more than twice the rate in the Swedish population in general [7].

The Danish Adoption Register [8] covers adoptions granted in 1924 to 1947, providing the opportunity to study cause-specific mortality in a broad age range from 22 to 90 years and consequently to analyse not only events early in life, but also causes of death which are typical later in life, such as vascular death causes. Our aim was to compare all-cause mortality rates in adoptees and the general population and to compare the cause-specific mortality for the following causes: infections, vascular causes, cancer, alcohol-related deaths and suicide.

## Materials and Methods

The study was based on the Danish Adoption Register, which contains records on all 14,425 non-familial adoptions formally granted in Denmark during the period 1924 through 1947. [8] The age at formal adoption was between 2 weeks and 22 years, the median age at adoption was 1 year. We excluded 272 adoptees born before 1917, 812 non-traceable adoptees, and 142 who were transferred to the adoptive family after the adoptee turned 7 years. For the period 1931–1970, the mortality rates of the adoptees were compared to the rates found in Statistical Year books and published as a whole by Andersen and coauthors [9]. The ages covered were 5 years to 90 years.

For the cause-specific analyses we identified all persons born in Denmark 1917 to 1947 and alive in 1970, in the Danish Civil Registration System (CRS) [10], comprising 1,920,326 individuals. The adoptees were also in the sample of the general population. The adoptees and the general Danish population were followed from 1970 and until death, emigration, or until censoring at 31 December 2006, whichever came first. The 36 years of follow up covered the ages between 22 to 90 years. The adoptees were compared to the general population born during the same period with regard to rate of death from all-causes as well as cause-specific death rates for the following causes: infections, vascular causes, cancer, alcohol-related deaths and suicide. For the cause-specific analyses, the primary/underlying cause of death was used, which was obtained from the Cause of Death Register.

In accordance with the possibilities for estimating the expected mortality, the final study sample included 13,111 adoptees: 6968 women and 6143 men, after excluding 88 dying before reaching 5 years of age. In the cause-specific analyses additionally 382 dying before 1970 were excluded.

### Statistical methods

Mortality rates for the general Danish population were available for men and women, in ten-year age groups (5–14, 15–24, 25–34, 35–44, 45–54, 54–64, 65–74, 75–84, 85-) and 10-year calendar periods (1931–1940, 1941–1950, 1951–1960, 1961–1970) [6].

Mortality rates and cause-specific mortality rates for the general Danish population born in 1917 to 1947 were calculated in men and women, in ten-year age groups (22–34, 35–44, 45–54, 54–64, 65–74, 75–84, 85-) and 10-year calendar periods (1971–1980, 1981–1990, 1991–2000, 2001–2006).

The rates in the Danish population and the person-years at risk in the adoptees were used within the strata of gender, age and calendar time to estimate expected number of deaths in the adoptees assuming the same rate of death as the general Danish population. The standardized mortality ratios (SMR) were calculated as the ratios between the observed and the expected number of deaths among the adoptees. Statistical significance was assessed based on the Poisson distribution. Analyses were carried out using Stata version 10.0 (StataCorp, College Station, TX).

### Ethics

Since the study is entirely based on register data, there was according to Danish law no request for an ethical permission. The study was approved by Danish Data Protection Agency.

## Results

**Figure 1:**
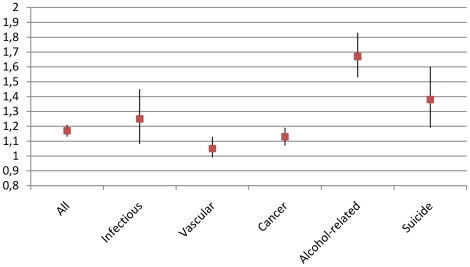
Excess mortality among Danish adoptees. Standardized Mortality Ratios in the period 1970 to 2006 of the 12,729 Danish adoptees with the general Danish population as reference. All death causes combined and for deaths with infections, vascular causes, cancer, alcohol-related deaths and suicides.

Comparing the 13,111 adoptees to all Danes from the same birth cohorts, the standardized mortality ratio (SMR) was 1.22 (95% CI 1.18–1.26) and when restricting to the ages before 65 years it was 1.30 (1.26–1.35). Among those surviving to 1970, we found that both the all-cause SMR and all of the cause-specific SMR were above 1.0 (see Figure 1). Statistically, the SMR was significantly above 1 due to infections, cancer, alcohol-related deaths, suicide and all-causes. No significant differences between the two sexes were found for the SMR (p>0.2). Gender specific SMR are presented in Table 1 for all deaths and for deaths before 65 years.

**Table 1:** Standardized Mortality Ratios in the period 1970 to 2006 of the 12,729 Danish adoptees with the general Danish population as reference.

		Men		Women	
Causes of death		Numbers of deaths	SMR	Numbers of deaths	SMR
**All-cause**	All	1930	1.17 (1.12–1.22)	1799	1.17 (1.12–1.23)
	≤65 years	1529	1.26 (1.19–1.33)	1218	1.27 (1.03–1.56)
**Infections**	All	80	1.23 (0.99–1.53)	90	1.27 (1.03–1.56)
	≤65 years	46	1.56 (1.20–2.02)	57	1.36 (1.02–1.82)
**Vascular**	All	530	1.05 (0.97–1.15)	362	1.06 (0.95–1.17)
	≤65 years	336	1.17 (1.05–1.30)	160	1.26 (1.08–1.47)
**Cancer**	All	579	1.11 (1.02–1.20)	754	1.15 (1.07–1.23)
	≤65 years	356	1.11 (1.00–1.23)	486	1.19 (1.09–1.30)
**Alcohol-related deaths**	All	344	1.62 (1.46–1.80)	163	1.79 (1.53–2.04)
	≤65 years	294	1.62 (1.45–1.82)	136	1.86 (1.57–2.20)
**Suicides**	All	104	1.36 (1.13–1.65)	72	1.41 (1.12–1.78)
	≤65 years	98	1.32 (1.08–1.62)	67	1.44 (1.13–1.83)

Numbers of deaths among the adoptees and gender specific SMR of all death causes combined and for deaths with infections, vascular causes, cancer, alcohol-related deaths and suicides.

In additional analyses, we estimated the excess mortality rate according to age and calendar time (Table 2). The excess mortality was significant in all calendar groups and in the age groups from 15–64 years. Including calendar time and age in the same model; the effect of age (p = 0.00001) was strong, whereas there was no significant effect of calendar time (p = 0.10).

**Table 2:** Standardized Mortality Ratios in the period 1926 to 2006 of the 13,111 Danish adoptees with the general Danish population as reference.

		Numbers of deaths	SMR
**All age groups in total**		4113	1.22 (1.18–1.26)
**Before age 65 years in total**		2747	1.30 (1.26–1.35)
**Age groups (years)**	5–15	66	0.91 (0.71–1.17)
	15–25	144	1.38 (1.17–1.62)
	25–34	149	1.27 (1.08–1.49)
	35–44	324	1.31 (1.18–1.46)
	45–54	751	1.33 (1.23–1.42)
	55–64	1313	1.31 (1.24–1.39)
	65–75	911	1.11 (1.04–1.18)
	75–89	455	1.04 (0.95–1.14)
**Calendar time**	1926–1940	29	1.02 (0.70–1.50)
	1941–1950	87	1.36 (1.10–1.68)
	1951–1960	95	1.18 (0.96–1.44)
	1961–1979	151	1.11 (0.95–1.31)
	1971–1980	357	1.30 (1.18–1.45)
	1981–1990	767	1.25 (1.16–1.34)
	1991–2000	1445	1.23 (1.17–1.30)
	2001–2006	1182	1.18 (1.12–1.25)

Numbers of deaths among the adoptees and SMR of all death causes combined in age groups and in calendar periods.

## Discussion

The adoptees have an excess mortality rate compared to the general Danish population across several major causes of death, including infectious diseases, vascular diseases, cancer, alcohol-related deaths and suicide. For all-causes combined, the excess is between 18 to 26%, and slightly higher for deaths between 15 and 65 years of age. In general the excess is higher before age 65 years, for vascular causes the excess is significant only when restricting to deaths before age 65 years. The largest excess was seen in alcohol-related deaths; 60% excess in men, and 80% in women.

In general, excess mortality in adoptees may be the result of an excess of risk factors in the biological family or a long-term effect of the adoption itself. The family factors in the causal chain resulting in adoption may include risk factors leading to long-term adverse health effects in the adoptee. These factors may include poor physical and mental health in the biological parents and associated genetic predisposition in the adoptee. Another factor is poverty and social problems causing suboptimal gestational conditions and thereby adversely predisposing the adoptee. Adopted children were typically born to young unmarried mothers and unplanned pregnancies may have been the most common immediate reason for adoption (in a subsample of Danish adoptees, examined by Eldred and co-authors, 82% of the biological mothers of adoptees were unmarried [11]). Unplanned pregnancy and single mother status may be associated with both psychological and social risk factors and there is some suggestion that having a biological father from a low social class is associated with increased mortality from natural causes in the adoptee [12], although the effect was small. The adoption process or the handling of disclosure of the adoption to the adoptee might cause long-term effects of the adoption itself leading to excess mortality. Depending on the specific circumstances, including timing of transfer, the adoption process may involve disruption of already formed attachment relationships and substantial changes in the social environment with possible long-term effects on personality development and social functioning [13]. Disclosure of the adoption may have psycho-pathogenic consequences, especially when revealed in adolescence or later, and when revealed by others than the adoptive parents [11].

Furthermore, the adoptive parents may also change behavior as a consequence of the adoption which might cause adoptive parents to be more open minded in their upbringing of the adoptee, e.g. in choice of career for the adoptee, and thereby lessen the familial influence [14]. Finally, lack of knowledge about the biological family background may also influence health and mortality in adoptees. For non-adoptees it may be possible to counteract what may appear to be a genetically based predisposition to disease by behavioral change (for example by quitting smoking because a parent was diagnosed with lung cancer) while lack of knowledge of family dispositions makes counteracting impossible in adoptees [1].

Our finding of between 32% and 44% excess risk of suicide may be compared with a similar study in a cohort of Swedish adoptees [7]. They were born in 1963 through 1973, were adopted by Swedish parents, and were followed from 1987 to 2002, and showed an estimated rate ratio of suicide was 2.2 (1.6–3.0) compared to non-adoptees [7]. The difference in excess rate of suicide in Danish and Swedish adoptees might partly be caused by the younger ages examined in the Swedish study. However, WHO reports the rate of suicide in the 1980'ies to be about 50% higher in Denmark than in Sweden, and, accordingly, the difference in the excess rate of suicide among adoptees in Denmark and Sweden might be explained by general population differences. Alternatively, the difference may be due to a gene-environmental interaction, which could be present in the Swedish study, since familial adoptions were not excluded.

The high rates of suicides and alcohol-related deaths may be due to genetic predisposition since genetic influence has been shown for both suicides [15], [16] and for alcohol abuse [17], [18]. On the other hand, the excess in suicide rates and alcohol-related deaths may also suggest that the higher mortality in adoptees partly reflects psychological issues. Although Teasdale and Owen found no differences between adoptees and non-adoptees with respect to achieved social class [14], it is evident that adoptees have an excess of mental and behavioral problems during childhood [4] and probably psychological problems later in life. The role of familial factors on rate of suicide in an adoption setting show conflicting results; in a Danish study, suicide rates have been found to be higher among adoptees in adoptive families from high or middle social classes compared to adoptive families from low social classes [12]. However in a large recent Swedish study there were indications of the opposite [16]. The origin of the excess of psychological problems may be genetic, due to prenatal factors, or the result of the early traumatic experience that adoption presumably is to at least some adoptees.

Previous Danish adoption studies have shown a genetic influence on mortality for infectious-, vascular causes and suicides, but not for cancer [1]–[3], [15]. Estimates of genetic and environmental influences are based on the associations between adoptees and biological and adoptive relatives. Consequently, the factors leading to excess mortality in adoptees will only affect these estimates if they have an impact on the associations, which is unlikely. However, the relative influence of genetic and environmental factors in the special setting of the adoption studies may be biased compared to the general population. Thus, a high load of risk factors in the biological family will not necessarily change the associations, which may directly reflect the genetic disposition. However, the relative influence of genetic and environmental factors in the special setting of adoptions studies may be baised compared to the general population. Besides special gene-environment interactions may influence the estimated associations. An example is that lack of the option of counteracting a known genetic predisposition might increase associations between adoptees and biological relatives, while psychological long-term effects of the adoption might decrease associations with both biological and adoptive relatives since these effects are specific to the adopted individual.

In conclusion, compared to the general population adoptees have an excess all-cause mortality. All major causes examined contributed to the excess mortality, and the excess was highest for alcohol-related deaths and suicide.
